# *Streptomyces* sp. MUM212 as a Source of Antioxidants with Radical Scavenging and Metal Chelating Properties

**DOI:** 10.3389/fphar.2017.00276

**Published:** 2017-05-17

**Authors:** Loh Teng-Hern Tan, Kok-Gan Chan, Tahir Mehmood Khan, Sarah Ibrahim Bukhari, Surasak Saokaew, Acharaporn Duangjai, Priyia Pusparajah, Learn-Han Lee, Bey-Hing Goh

**Affiliations:** ^1^Novel Bacteria and Drug Discovery Research Group, School of Pharmacy, Monash University MalaysiaBandar Sunway, Malaysia; ^2^Division of Genetics and Molecular Biology, Institute of Biological Sciences, Faculty of Science, University of MalayaKuala Lumpur, Malaysia; ^3^Department of Pharmacy, Abasyn UniversityPeshawar, Pakistan; ^4^Department of Pharmaceutics, College of Pharmacy, King Saud UniversityRiyadh, Saudi Arabia; ^5^Center of Health Outcomes Research and Therapeutic Safety, School of Pharmaceutical Sciences, University of PhayaoPhayao, Thailand; ^6^Pharmaceutical Outcomes Research Center, Faculty of Pharmaceutical Sciences, Naresuan UniversityPhitsanulok, Thailand; ^7^Unit of Excellence on Herbal Medicine, School of Pharmaceutical Sciences, University of PhayaoPhayao, Thailand; ^8^Division of Physiology, School of Medical Sciences, University of PhayaoPhayao, Thailand; ^9^Jeffrey Cheah School of Medicine and Health Sciences, Monash University MalaysiaBandar Sunway, Malaysia

**Keywords:** *Streptomyces* sp., Malaysia, mangrove, antioxidant, radical-scavenging

## Abstract

Reactive oxygen species and other radicals potentially cause oxidative damage to proteins, lipids, and DNA which may ultimately lead to various complications including mutations, carcinogenesis, neurodegeneration, cardiovascular disease, aging, and inflammatory disease. Recent reports demonstrate that *Streptomyces* bacteria produce metabolites with potent antioxidant activity that may be developed into therapeutic drugs to combat oxidative stress. This study shows that *Streptomyces* sp. MUM212 which was isolated from mangrove soil in Kuala Selangor, Malaysia, could be a potential source of antioxidants. Strain MUM212 was characterized and determined as belonging to the genus *Streptomyces* using 16S rRNA gene phylogenetic analysis. The MUM212 extract demonstrated significant antioxidant activity through DPPH, ABTS and superoxide radical scavenging assays and also metal-chelating activity of 22.03 ± 3.01%, 61.52 ± 3.13%, 37.47 ± 1.79%, and 41.98 ± 0.73% at 4 mg/mL, respectively. Moreover, MUM212 extract was demonstrated to inhibit lipid peroxidation up to 16.72 ± 2.64% at 4 mg/mL and restore survival of Vero cells from H_2_O_2_-induced oxidative damages. The antioxidant activities from the MUM212 extract correlated well with its total phenolic contents; and this in turn was in keeping with the gas chromatography–mass spectrometry analysis which revealed the presence of phenolic compounds that could be responsible for the antioxidant properties of the extract. Other chemical constituents detected included hydrocarbons, alcohols and cyclic dipeptides which may have contributed to the overall antioxidant capacity of MUM212 extract. As a whole, strain MUM212 seems to have potential as a promising source of novel molecules for future development of antioxidative therapeutic agents against oxidative stress-related diseases.

## Introduction

Recently, the discovery of novel bioactive molecules and properties of microorganisms from mangrove environment have led to great interest in exploiting these organisms; which have already contributed immensely through industrial and clinical applications (Mehta and Satyanarayana, 2016; Romano et al., 2016; Tan et al., 2016). Among these novel mangrove derived *Streptomyces* species with bioactive potentials isolated recently include *S. pluripotens* (Lee et al., 2014b), *S. mangrovisoli* (Ser et al., 2015b), *S. gilvigriseus* (Ser et al., 2015c), *S. malaysiense* (Ser et al., 2016b), and *S. antioxidans* (Ser et al., 2016c). The bacterial genus *Streptomyces* represents the largest genus of *Actinobacteria*, and since it was first proposed by Waksman and Henrici (1943) it has expanded to more than 780 species and 30 subspecies with validly published names^1^ at the time of writing (December 2016). The genus *Streptomyces* has made significant contributions to mankind by virtue of its innate capability of producing a wide range of bioactive compounds which confer its diverse biological activities (Bérdy, 2005; Lucas et al., 2013; Jauri et al., 2016). At the present time, more than 7,000 bioactive compounds with various key clinical applications have been discovered from *Streptomyces*, including drugs with antimicrobial, antioxidant, anticancer, antifungals and immunosuppressant properties (Bérdy, 2005, 2012; Karam and Wali, 2015). To date, bioprospecting of *Streptomyces* has facilitated the discovery of antibiotics such as streptomycin and erythromycin (Schatz et al., 1944; Weber et al., 1985), anticancer agents such as doxorubicin (Grimm et al., 1994) and bleomycin (Du et al., 2000), antifungals such as nystatin (Brautaset et al., 2000). These discoveries serve to highlight the enormous clinical impact *Streptomyces* derived drugs have already had on clinical medicine and the potential for new drug discoveries through exploration of novel species.

Reactive oxygen species (ROS) are constantly during oxygen-dependent aerobic metabolism, placing cells under continuous threat of oxidative damage. At low and moderate concentrations, ROS play an important role as signaling molecules involved in mitogenesis (Irani, 2000) or during infections as a host defense mechanism (Torres et al., 2006). However, disproportionately high generation of ROS creates sustained environmental stress within the intracellular milieu resulting in oxidative tissue damage. This condition is known as oxidative stress which is potentially dangerous as it can cause alteration to cell structure and function as well as inducing somatic mutations or DNA damage, leading to lipid and protein modifications and ultimately increased risk of neoplastic transformation (Dizdaroglu, 2015). Consequently, oxidative stress is strongly associated with many human diseases including cancer, diabetes (Giacco and Brownlee, 2010), cardiovascular (Fearon and Faux, 2009), and neurodegenerative diseases (Barnham et al., 2004).

In order to mount a defense against the damage caused by oxidants, cells, or organisms make use of antioxidants. ‘Antioxidant’ is the term for any compound that is able to block or delay the oxidative damage caused by the oxidants via giving electrons and/or hydrogen atoms and thus halting the chain reactions (Apak et al., 2016). Effective antioxidant activity can occur through one or more of several possible pathways such as preventing the formation of free radicals, disrupting the autoxidation chain reaction, quenching the singlet oxygen, reducing the ROS into stable compounds, chelating metal prooxidants, modulating other antioxidant enzymes and inhibiting pro-oxidative enzymes (Carocho and Ferreira, 2013; Zhang and Tsao, 2016). Endogenously, the formation of free radicals can be prevented and also neutralized by the antioxidant enzymes present in the cells such as glutathione peroxidase, catalase and the superoxide dismutase (SOD) enzymes. Despite their efficiency in the maintenance of free radical concentrations at low levels, depending solely on these endogenous antioxidant systems is insufficient to counter oxidative stress and humans therefore require input of various types of exogenous antioxidants that can be obtained through dietary intake and supplements (Carocho and Ferreira, 2013). Fruits and vegetables are an important part of a well-balanced and healthy diet for humans owing to their rich antioxidant content (Wong et al., 2012; Tan et al., 2015a). The consumption of an antioxidant-rich diet has been shown to well-related epidemiologically with a reduced risk of developing cardiovascular diseases and certain types of cancers (van’t Veer et al., 2000; Bazzano et al., 2002). Thus far, food sources have been the main focus of natural antioxidants (Tang et al., 2016); however, they are also found abundantly in metabolites produced by microbes (Juan and Chou, 2010; Rakesh et al., 2011; Sadrati et al., 2013). For instance, recent evidence documented that naturally antioxidative agent-producing strains of *Streptomyces* bacteria have been isolated from mangrove environments (Rao and Rao, 2013; Ser et al., 2015b; Tan et al., 2015b).

Given that *Streptomyces* species derived from the untapped mangrove ecosystem are believed to produce unique secondary metabolites with interesting bioactivities (Ser et al., 2015b; Tan et al., 2015b), this study aimed to evaluate the bioactive potential of a *Streptomyces* sp. MUM212 isolated from mangrove soil in Kuala Selangor, Malaysia including its antioxidant properties. Several *in vitro* antioxidant assays were employed to evaluate the antioxidant potentials of MUM212 extract. The potential of MUM212 extract in protecting cells from oxidative stress was also evaluated using a hydrogen peroxide (H_2_O_2_)-induced oxidative stress model. Additionally, the chemical constituents present in MUM212 extract were determined through gas chromatography and mass spectrometry analysis. It was found that the chemical constituents detected from MUM212 extract correlated well with the antioxidant properties demonstrated by the extract. In short, the findings of this study provide a strong push toward further exploration of mangrove *Streptomyces* as a source of potential antioxidative agents, particularly with a view toward the development of drugs with therapeutic as well as preventive applications against oxidative stress related diseases.

## Materials and Methods

### Isolation and Preservation of Pure Culture MUM212 Strain

In Jan 2015, the pure culture of MUM212 strain was isolated from a mangrove soil sample obtained from the designated site MUM-KS1 (3° 21′ 45.8″ N 101° 18′ 4.5″ E) at Kuala Selangor, Malaysia. The soil sample was collected within the layer of top 20 cm after removing the top 2–3 cm layer of soil and kept in sterile plastic bags at -20°C prior to processing. After being air dried, the soil sample was ground and subjected to pretreatment with wet heat method based on Takahashi et al. (1996). Prior to spread plating onto the isolation medium ISP2, the pretreated air-dried soils were diluted with sterilized water (Shirling and Gottlieb, 1966). The isolation medium ISP2 was prepared aseptically and supplemented with antifungal agents: cycloheximide (25 μg/ml) and nystatin (10 μg/ml). The inoculated ISP2 agar plate was incubated at 28°C for 14 days before the isolation of pure culture of strain MUM212. Strain MUM212 was purified with new ISP2 agar and maintained on slants of ISP2 agar at 28°C and preserved in 20% (v/v) glycerol suspensions at -20°C.

### 16S rRNA PCR and Phylogenetic Analyses

**FIGURE 1:**
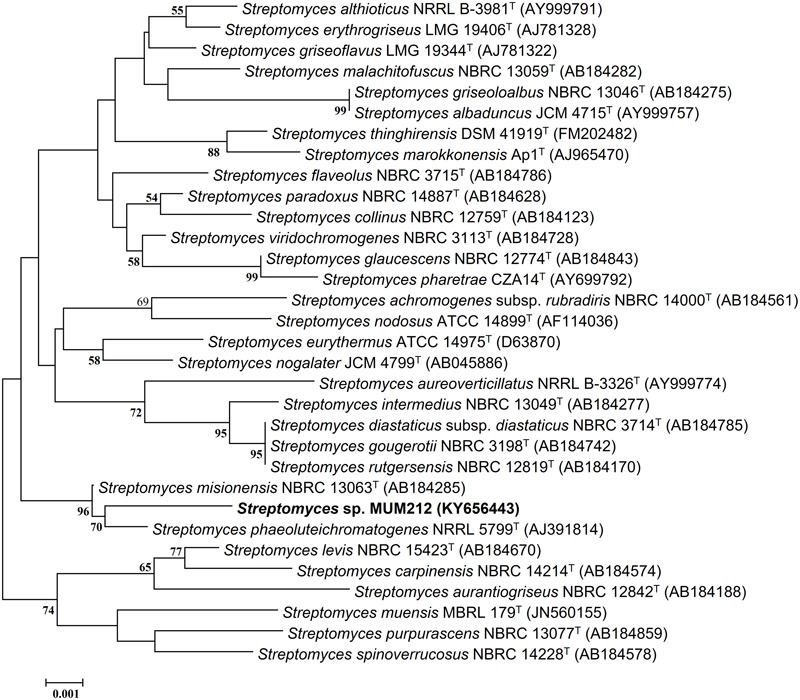
**Neighbor-joining phylogenetic tree based on almost complete 16S rRNA sequences (1341 nucleotides) showing relationship between strain MUM212 and representatives of some other related taxa.** Bootstrap values (>50%) based on 1000 re-sampled datasets are shown at branch nodes. Bar: 0.001 substitutions per site.

The genomic DNA (gDNA) extraction of strain MUM212 was performed according to the method described in Hong et al. (2009). The extracted gDNA of strain MUM212 was subjected to 16S rRNA gene amplification based on protocol by Lee et al. (2014b). The primer pair 27F-1492R was used for the PCR amplification (Lee et al., 2014c). The sequences of the primer pair are as follows: 27F (5′-GTTTGATCCTGGCTCAG-3′), 1492R (5′-TACGGCTACCTTGTTACGACTT-3′). The PCR reactions involved a final reaction of 50 μl following manufacturer’s protocol (SolGent^TM^, South Korea) and the use of Kyratex PCR Supercycler (Kyratec, Australia) with the optimized cycling conditions: (i) 95°C for 5 min, (ii) 35 cycles of 94°C for 50 s, 55°C for 1 min and 72°C for 1 min 30 s; and (iii) 72°C for 8 min. The sequenced 16S rRNA gene of strain MUM212 was aligned with representative gene sequences of related type strains of the genus *Streptomyces* retrieved from the GenBank/EMBL/DDBJ databases using CLUSTAL-X software (Thompson et al., 1997). Phylogenetic trees were constructed with the neighbor-joining (Saitou and Nei, 1987) (**Figure 1**) algorithms using MEGA version 6.0 (Tamura et al., 2013). The evolutionary distances for the neighbor-joining algorithm were computed using Kimura’s two-parameter model (Kimura, 1980). The EzTaxon-e server^2^ (Kim et al., 2012) was used for calculations of sequence similarity. The stability of the resultant trees topologies were evaluated by using the bootstrap based on 1000 resampling method of Felsenstein (1985).

### Phenotypic Characteristics

In order to examine the morphology and cultural characteristics of strain MUM212, pure culture of MUM 212 was incubated for 14 days at 28 °C on a series of agar: International *Streptomyces* Project (ISP) 2, ISP3, ISP4, ISP5, ISP6, ISP7 (Shirling and Gottlieb, 1966), actinomycetes isolation agar (AIA) (Atlas, 1993), starch casein agar (SCA) (Küster and Williams, 1964), and nutrient agar (Mac Faddin, 1976). The colony color of strain MUM212 was determined based on ISCC-NBS color charts (Kelly, 1964). The 7–14 days culture of strain MUM212 was evaluated under both light microscopy (80i, Nikon) and scanning electron microscopy (TM-1000, Hitachi). The growth characteristics such as temperature tolerance, NaCl tolerance and pH tolerance of strain MUM212 were evaluated. In the temperature tolerance test, strain MUM212 was incubated at different temperatures ranging from 4 to 40°C at intervals of 4°C on ISP2 agar. For the NaCl tolerance test, strain MUM212 was grown in tryptic soy broth (TSB) supplemented with different salt concentrations ranging from 0 to 10% (w/v) at intervals of 2%. For the pH tolerance test, strain MUM212 was grown in TSB between pH2 and 10 at intervals of 1 pH unit. These tolerance tests were performed for 14 days. The ISP7 agar media was used to examine the productivity of melanoid pigments. The catalase activity was determined as described by Lee et al. (2014a). Hemolytic activity was evaluated using blood agar consisting of 5% (w/v) peptone, 3% (w/v) yeast extract, 5% (w/v) NaCl, and 5% (v/v) horse blood (Carrillo et al., 1996). The enzymatic activities in the digestion of amylase, cellulase, chitinase, lipase, protease, and xylanase were examined on ISP2 agar (Meena et al., 2013). Antibiotic susceptibility of MUM212 strain was evaluated by the disk diffusion method as described by Shieh et al. (2003). The antimicrobial disks and their concentrations per disk (Oxoid, Basingstoke, UK) were as follows: ampicillin (10 μg), ampicillin sulbactam (30 μg), cefotaxime (30 μg), chloramphenicol (30 μg), erythromycin (15 μg), gentamicin (20 μg), nalidixic acid (30 μg), penicillin G (10 μg), tetracycline (30 μg), and vancomycin (30 μg). The carbon-source utilization and chemical sensitivity assays were examined using Biolog GenIII MicroPlates (Biolog, United States).

### Extract Preparation of Strain MUM212

The fermentation process was initiated by inoculating 14 days culture of strain MUM212 into the fermentation medium. Han’s Fermentation Media 1 (HFM1) (Biomerge, Malaysia) was the selected fermentation medium for strain MUM212 (Hong et al., 2009; Lee et al., 2012). The fermentation process was performed in an Erlenmeyer flask containing the HFM1 for 7–10 days at 28°C with shaking speed set at 200 rpm. The biomass was removed by centrifugation at 12000 × *g* for 15 min while the supernatant was collected by filtration with filter paper (Whatman, UK). The supernatant was freeze-dried and extracted with methanol for 72 h. The methanol-containing extract was filtered and subjected to rotary evaporation to remove the methanol from the extract at 40°C. The extract was collected and dissolved in dimethyl sulfoxide (DMSO) prior to further analysis.

### Antioxidant Capacity of MUM212 Extract

#### DPPH-Radical Scavenging Activity

DPPH (2,2-diphenyl-1-picrylhydrazyl) radical scavenging activity of MUM212 extract was examined based on a previously described method with minor changes (Ser et al., 2015b). MUM212 extract at varying concentrations was reacted with freshly prepared 0.016% (w/v) DPPH in 95% (v/v) ethanol. The reaction was left in the dark at room temperature for 20 min prior to the measurement of absorbance at 515 nm with a microplate reader. Gallic acid was utilized as the positive control. The percentage DPPH radical scavenging activity was computed according to the following formula:

$$\%DPPH scavenging activity=\frac{Absorbance of control-Absorbance of sample}{Absorbance of control}\times 100\%$$

#### Superoxide Anion Scavenging Activity

The superoxide anion scavenging activity or SOD like activity of MUM212 strain was examined using an indirect colorimetric method (19160 SOD Assay Kit-WST, Sigma Aldrich) that measures the formation of water soluble formazan dye upon the reduction of [2-(4-iodophenyl)-3-(4-nitrophenyl)-5-(2,4-disulfophenyl)-2H-tetrazolium, monosodium salt] (WST-1) by superoxide anion. In short, a series of different concentrations of MUM212 extract were loaded into respective wells of a 96-well plate before the addition of the respective reaction solutions based on the manufacturer’s protocol. It was then followed by measurement of absorbance at 450 nm using a microplate reader after incubation at 37°C for 30 min to measure the SOD-like activity of MUM212 extract. The superoxide anion scavenging activity or SOD-like activity was calculated according to the formula expressed below:

$$\%SOD like activity\;=\frac{\begin{array}{c} (\mathrm{Abs}\;\mathrm{control}\;\mathrm{blank}\;-\;\mathrm{Abs}\;\mathrm{buffer}\;\;\mathrm{blank})\;-\\ \left(\mathrm{Abs}\;\mathrm{sample}\;-\;\mathrm{Abs}\;\mathrm{sample}\;\mathrm{blank}\right) \end{array}}{\begin{array}{c} \mathrm{Abs}\;\mathrm{control}\;\mathrm{blank}\;-\;\mathrm{Abs}\;\mathrm{buffer}\;\mathrm{blank} \end{array}}\times 100\%$$

Abs, absorbance measured at 450 nm.

#### ABTS Radical Scavenging Activity

The 2,2′-azino-*bis*(3-ethylbenzothiazoline-6-sulfonic acid) (ABTS) radical scavenging assay was conducted according to Ser et al. (2016c). Prior to the assay, ABTS stock solution at 7 mM was mixed with potassium persulfate at 2.45 mM to generate ABTS radical cation (ABTS∙+) for 24 h. The ABTS radical solution was mixed with MUM212 extract of varying concentrations preloaded in a 96-well microplate. The reaction was left to proceed in the dark at room temperature for 20 min before the measurement of absorbance at 743 nm with a microplate reader. Gallic acid was used as a positive control. The percentage ABTS scavenging activity was indicated by the reduction in the absorbance of ABTS radical and was computed using the formula expressed below:

$$\%ABTS scavenging activity=\frac{Absorbance of control-Absorbance of sample}{Absorbance of control}\times 100\%$$

#### Metal Chelating Activity

Metal chelating activity of MUM212 extract was measured as described by Adjimani and Asare (2015). The assay measures the color reduction in the presence of chelators that disrupt the formation of red ferrous ion and ferrozine complexes. Briefly, FeSO_4_ at 2 mM was added into a microplate preloaded with MUM212 extract at a series of concentrations. After that, ferrozine at 5 mM was added to initiate the reaction and left to incubate for 10 min at room temperature. The absorbance of the mixtures were determined at a wavelength of 562 nm. EDTA was used as a positive control. The metal chelating activity or percentage inhibition of ferrozine-Fe^2+^ complex formation was computed based on the formula expressed below:

$$\%Metal chelating activity\;=\;\frac{\mathrm{Absorbance}\;\mathrm{of}\;\mathrm{control}\;-\;\mathrm{Absorbance}\;\mathrm{of}\;\mathrm{sample}}{\mathrm{Absorbance}\;\mathrm{of}\;\mathrm{control}}\;\times \;100\%$$

### Lipid Peroxidation Assay

A modified thiobarbituric acid reactive species (TBARS) assay was employed to measure the inhibitory potential of MUM212 extract against lipid peroxidation using egg yolk homogenate as lipid-rich media, as described by Dasgupta and De (2004). Malondialdehyde (MDA), a secondary end product of oxidation of polyunsaturated fatty acids forms TBA-MDA adduct, with maximal excitation at 535 nm and emission at 553 nm, upon reaction with two molecules of thiobarbituric acid (TBA). Briefly, 800 μL egg homogenate (10% in phosphate buffered saline, v/v) was mixed with increasing concentrations of extract and incubated at 37°C for 1 h in the presence of 100 μM of FeSO_4_ to induce lipid peroxidation. Thereafter, the reaction was stopped by adding ice-cold 20% trichloroacetic acid (1:1) to the incubates prior to centrifugation at 1,200 × *g* for 10 min. The extent of lipid peroxidation was determined by measuring the MDA content in the sample supernatants using TBARS. One milliliter of TBARS reagent [0.8% TBA (w/v), 10 mL of 20% acetic acid and pH adjusted to 3.5 with NaOH] was added to 500 μL of sample and heated at 95°C for 1 h. After cooling on ice, the product was measured by fluorometer at 535 excitation/553 nm emission. Inhibition of the lipid peroxidation (%) by the extract was calculated with the formula: % inhibition of lipid peroxidation = $\left(\frac{RFI_{\mathrm{Blank}}-RFI_{\mathrm{Sample}}}{RFI_{\mathrm{Blank}}}\right)$ x 100, RFI, relative fluorescence intensity; Blank, without extract.

### *In Vitro* Cytoprotective Assay

#### Cell Culture and Treatment

Vero cell line from American Type Culture Collection (ATCC) were cultured in Roswell Park Memorial Institute (RPMI) 1640 medium supplemented with 10% fetal bovine serum and 1x antibiotic-antimycotic (Gibco). The cells were maintained at 37°C in a humidified incubator containing 5% CO_2_.

Before treatment, cells were seeded in 96-well plates at a density of 10,000 cells/well and cultured overnight. In all experiments, the cells were pre-incubated with increasing concentrations of MUM212 extract for 2 h before treatment with 350 μM of H_2_O_2_ for 24 h. The extract was prepared in DMSO and maintained at a final concentration of 0.5% (v/v) for all experiments. The control group was treated in the presence of 0.5% (v/v) DMSO under the same culture conditions.

#### Measurement of Cell Viability (MTT Assay)

The cell survival was evaluated using 3-(4,5-dimethylthiazol-2-yl)-2,5-diphenyltetrazolium bromide (MTT) assay as described by (Goh and Kadir, 2011) with minor modifications. The MTT assay is a colorimetric assay that measures the mitochondrial activity of the viable cells. After treatment (see Cell Culture and Treatment), MTT (Sigma) was added to each well and incubated at 37°C in a humidified atmosphere with 5% CO_2_ for 4 h. After complete aspiration of the medium from the wells, 200 μL of DMSO was added to dissolve the formazan crystals. The absorbance of dissolved formazan product was measured spectrophotometrically at 570 nm using a microplate reader. The percentage of cell viability was calculated as follows:

$$Percentage of cell viability=\frac{Absorbance of treated cells}{Absorbance of untreated cells(0.5\%DMSO only)}\times 100\%$$

### Total Phenolic Content Determination with Folin–Ciocalteu’s Reagent Method

The total phenolic content (TPC) in MUM212 extract was estimated with Folin–Ciocalteu’s reagent method in 96-well plates adapted from Zhang et al. (2006) with slight modification. Briefly, 10 μL of samples were mixed with 50 μL of (1:10) diluted Folin–Ciocalteu’s reagent. After 5 min of incubation in the dark, 40 μL of 7.5% sodium carbonate was added into each well-followed by 30 min incubation at room temperature. The absorbance of each well was measured with a microplate reader at 750 nm and the results were expressed in gallic acid equivalents (GAEs).

### Flavonoid Content Determination Based on Aluminum–Flavonoid Complexes Formation Method

The total flavonoid content in MUM212 extract was quantified using the spectrophotometric 96 well-microplate method described by Herald et al. (2012) with minor modification. Firstly, 100 μL of distilled water was added into each of the 96 wells, followed by 10 μL of 50 g/L NaNO_2_ and 25 μL of standard or extract. After 5 min, 15 μL of 100 g/L AlCl_3_ was added to the mixture. Next, 50 μL of 1 M NaOH and 50 μL of distilled water were added into all the wells after 6 min of incubation. The plate was shaken for 30 s in the plate reader prior to absorbance measurement at 510 nm. Catechin was used as the standard.

### Gas Chromatography–Mass Spectrometry (GC–MS) Analysis

Gas chromatography-mass spectrometry (GC–MS) analysis was conducted based on our previously developed protocol with slight modifications (Supriady et al., 2015). The Agilent Technologies 6980N (GC) equipped with 5979 Mass Selective Detector (MS), HP-5MS (5% phenyl methyl siloxane) capillary column of dimensions 30.0 m × 250 μm × 0.25 μm and helium as carrier gas at 1 mL/min were used for the analysis. The column temperature was maintained initially at 40°C for 10 min, followed by an increase of 3°C/min to 250°C and was kept isothermal for 5 min. The MS was operating at 70 eV. The constituents were identified by comparison of their mass spectral data with those available from W9N11 MS library.

### Statistical Analysis

The antioxidant assays were performed in quadruplicate. Results were expressed in mean ± standard deviation (SD). SPSS software was used to perform the statistical analysis. One-way analysis of variance (ANOVA) and Tukey’s *post hoc* analysis were used to determine whether there was a significant difference between the treated and untreated groups. A difference was considered statistically significant when *p* ≤ 0.05. Correlation analysis was also performed using SPSS to determine the relationship between the phenolic content and the antioxidant capacity of the extract.

## Results

### 16S rRNA PCR and Phylogenetic Analyses

The sequencing result revealed the nearly complete 16S rRNA gene sequence of MUM212 was 1341 bp (GenBank/EMBL/DDBJ accession number KY656443). The 16S rRNA gene sequence of strain MUM212 was aligned with the corresponding partial 16S rRNA gene sequences of the type strains of representative members retrieved from GenBank/EMBL/DDBJ databases. A phylogenetic tree constructed based on the 16S rRNA gene sequences showed that strain MUM212 (**Figure 1**) formed a distinct clade with type strain *S. phaeoluteichromatogenes* NRRL5799^T^ at bootstrap value of 70%, indicating the high confidence level of the association (**Figure 1**). Furthermore, strain MUM212 showed highest 16S rRNA sequence similarity to that of *S. misionensis* NBRC13063^T^ (99.5%) and followed by *S. phaeoluteichromatogenes* NRRL5799^T^ (99.4%).

### Phenotypic Analyses of Strain Streptomyces sp. MUM212

**FIGURE 2:**
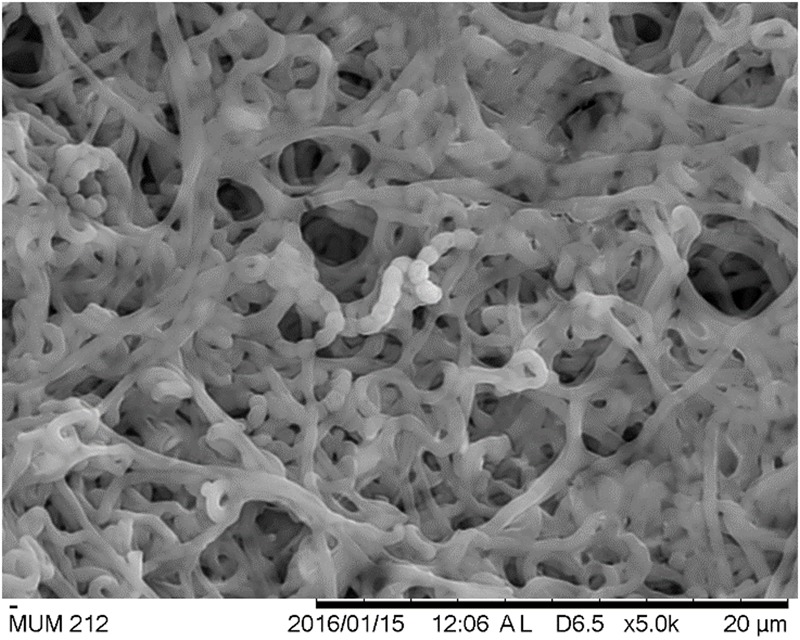
**The scanning electron micrographs of strain MUM212.** It appear as filaments and branch to form a network of filaments called mycelium. Spiral shape spore chains are also clearly visible in SEM micrographs.

Strain MUM212 is Gram-positive and aerobic. The colony color of aerial and substrate mycelium on different solid media are shown in **Table 1** (Supplementary Figure S1). The strain grows well on both ISP2 and ISP3 agar but only moderately on ISP5, ISP7 agar, AIA, nutrient agar and SCA after 1–2 weeks at 28°C. However, strain MUM212 does not grow on ISP4 agar and grow poorly on ISP6 agar. On ISP2 agar, it grows abundantly in both aerial and vegetative hyphae. Furthermore, strain MUM212 produces spiral spore chains observed using the scanning electron microscopy (**Figure 2**). Strain MUM212 is positive for catalase but negative for both hemolytic activity and melanoid pigment production. These phenotypic characteristics of strain MUM212 are in line with the genus *Streptomyces* as described in Whitman et al. (2012) in that they are aerobic, Gram positive, catalase-positive, bacteria that form extensively branched substrate and aerial mycelia. Most of the *Streptomyces* species grow optimally at the temperature range of 25–35°C and pH range of 6.5–8.0 (Whitman et al., 2012). Indeed, strain MUM212 also grows between pH 4 and 7 (optimum at pH 7), 0–6% of NaCl concentration (optimum 2%) and 20 to 40°C (optimum at 32°C).

**Table 1:** The colony characteristics of strain MUM212.

	Color of colony mycelium			Color of colony mycelium	
Media	Aerial	Substrate	Media	Aerial	Substrate
ISP2	Vivid yellow	Moderate yellow	ISP3	Dark grayish yellow	Grayish greenish yellow
ISP5	Greenish white	Pale greenish yellow	ISP6	Pale yellowish green	Light greenish yellow
ISP7	Yellowish white	Pale yellow	AIA	Light yellow	Yellowish white
SCA	Greenish white	Light yellow	NA	Yellowish white	Pale yellow

In the enzymatic activity test, strain MUM212 was shown to digest soluble starch and carboxymethylcellulose, indicating it produces amylase and cellulase enzymes respectively (Supplementary Figure S2). However, strain MUM212 does not degrade tributyrin, xylan, casein, and chitin. Strain MUM212 was found to be able to utilize various compounds as carbon sources (**Table 2**). Furthermore, chemical sensitivity tests showed that the strain was resistant to 1% sodium lactate, rifamycin RV, minocycline, guanidine HCl, Niaproof 4, tetrazolium violet, tetrazolium blue, potassium tellurite, aztreonam, sodium butyrate, and sodium bromate. For the antibiotic sensitivity test, it was sensitive to ampicillin, ampicillin sulbactam, cefotaxime, chloramphenicol, erythromycin, gentamicin, Penicillin G, tetracycline, and vancomycin. Strain MUM212 is only resistant to nalidixic acid.

**Table 2:** The utilization of carbon and nitrogen sources by strain MUM212.

Utilizable carbon and nitrogen sources		Non-utilizable carbon and nitrogen sources	
Acetic acid	Acetoacetic acid	3-Methyl glucose	β-Methyl-D-glucoside
α-D-Glucose	α-D-Lactose	Bromo-succinic acid	D-Aspartic acid
α-Hydroxy-butyric acid	α-Keto-butyric acid	D-Fucose	D-Sorbitol
α-Keto-glutaric acid	β-Hydroxy-D,L-butyric acid	D-Turanose	L-Fucose
Citric acid	D-Arabitol	L-Lactic acid	L-Rhamnose
D-Cellobiose	Dextrin	Mucic acid	*N*-acetyl -*b*-D-mannosamine
D-Fructose	D-Fructose-6-PO_4_	*N*-acetyl-D-galactosamine	*N*-acetyl-neuraminic acid
D-Galactose	D-Galacturonic acid	*p*-hydroxy-phenylacetic acid	Quinic acid
D-Gluconic acid	D-Glucose-6-PO_4_	Stachyose	Sucrose
D-Glucuronic acid	D-Lactic acid methyl ester		
D-Malic acid	D-Maltose		
D-Mannitol	D-Mannose		
D-Melibiose	D-Raffinose		
D-Saccharic acid	D-Salicin		
D-Serine	D-Trehalose		
Formic acid	γ-Amino -butyric acid		
Gelatin	Gentiobiose		
Glucuronamide	Glycerol		
Glycyl-L-proline	Inosine		
L-Alanine	L-Arginine		
L-Aspartic acid	L-Galactonic acid lactone		
L-Glutamic acid	L-Histidine		
L-Malic acid	L-Pyroglutamic acid		
L-Serine	Methyl pyruvate		
Myo-inositol	*N*-acetyl-D-glucosamine		
Pectin	Propionic acid		
Tween 40			

*The carbon and nitrogen sources utilization of strain MUM212 was assessed using the Biolog GEN III Microplate. The positive utilization of certain carbon source is indicated by the formation of purple color in the 96 wells of Microplate, thereby the increased respiration as result of the growth of strain MUM212 causes reduction of the tetrazolium redox dye pre-loaded in the well. Meanwhile, negative utilization or no growth of strain MUM212 is indicated by the colorless well.*

### Antioxidant Activity

In this study, we investigated the antioxidant potential of MUM212 extract based on several *in vitro* antioxidant assays including the DPPH radical scavenging activity, ABTS radical scavenging activity, SOD-like activity and metal-chelating activity assays. The results these assays are tabulated in **Table 3**. DPPH is a simple and robust antioxidant activity screening assay. The DPPH assay is used to assess the free radical scavenging ability of a substance/compound by using a stable free DPPH radical. A substance/compound which can transfer hydrogen atoms or electron to the DPPH radicals results in the loss of the violet color of the DPPH radical. This is because it is the delocalization of the spare electron over the DPPH molecule that gives rise to its deep violet color (Molyneux, 2004). This study demonstrated that MUM212 extract exhibited DPPH radical scavenging activity based on the color changes observed from the violet DPPH radical solution into yellow-colored diphenylpicrylhydrazine (reduced form). The result showed that MUM212 extract exhibited significant DPPH scavenging activity measured from 5.80 ± 1.27 to 22.03 ± 3.01% of DPPH radicals reduction (*p* < 0.05) at doses ranging from 0.5 to 4 mg/mL, suggesting that MUM212 extract exhibits hydrogen donating ability.

**Table 3:** The antioxidant activities demonstrated by MUM212 extract in different antioxidant assays.

Concentration of MUM212 extract (μg/mL)	Antioxidant activities			
	DPPH radical scavenging activity (%)	ABTS radical scavenging activity (%)	Superoxide dismutase-like activity (%)	Metal-chelating activity (%)
250	0.47 ± 1.00	9.57 ± 1.32^∗^	17.78 ± 3.85^∗^	9.17 ± 1.16^∗^
500	5.80 ± 1.27^∗^	18.02 ± 1.14^∗^	23.24 ± 3.59^∗^	16.79 ± 1.75^∗^
1000	12.03 ± 0.88^∗^	27.00 ± 1.40^∗^	30.69 ± 2.35^∗^	25.01 ± 1.54^∗^
2000	20.00 ± 1.42^∗^	43.07 ± 2.36^∗^	33.66 ± 6.48^∗^	28.17 ± 3.28^∗^
4000	22.03 ± 3.01^∗^	61.52 ± 3.13^∗^	37.47 ± 1.79^∗^	41.98 ± 0.73^∗^

*^∗^Statistically significance (*p* < 0.05) when compared to control (without extract). ND, not detected; NT, not tested.*

The ABTS assay was another antioxidant assay used to evaluate the radical scavenging activity of MUM212 extract. This assay involves the use of ABTS∙+, a stable radical cation that can be generated chemically by reacting ABTS with potassium persulfate. Thermodynamically, the ABTS∙+ can be reduced by compounds which have a lower redox potential than that of ABTS (0.68 V). For example, the alcohols, monophenols, and amino acids present in the natural products have low redox potentials that can thus react with ABTS∙+ (Campos and Lissi, 1997; Apak et al., 2016). This assay revealed that the addition of MUM212 extract decolorized the intensely blue-green ABTS∙+ solution in a dose-dependent manner, suggesting that the MUM212 extract is capable of reducing the blue-green color of ABTS∙+ back into ABTS, which is colorless. The results showed that MUM212 extract exhibited significant ABTS radical scavenging activity (*p* < 0.05) measuring from 9.57 ± 1.32 to 61.52 ± 3.13% at concentrations ranging from 0.25 to 4 mg/mL.

MUM212 extract was also tested for its scavenging capabilities, particularly against superoxide anion radical (O_2_^∙-^) which is the most crucial ROS as it can give rise to several other forms of reactive oxygen intermediates (Bhattacharyya et al., 2014). In this study, the ability of MUM212 extract to scavenge the O_2_^∙-^ was investigated using WST-1 as a superoxide detector for the measurement of SOD-like activity (Peskin and Winterbourn, 2000). The assessment of the SOD-like activity of the extract was performed using a hypoxanthine-xanthine oxidase system as the source of O_2_^∙-^ coupled with WST-1, a tetrazolium salt that detects superoxide radicals and results in the production of highly water soluble WST formazan dye upon reduction by O_2_^∙-^ (Peskin and Winterbourn, 2000). The results showed that MUM212 extract exhibited significant SOD-like activity (*p* < 0.05) measuring from 17.78 ± 3.85 to 37.47 ± 1.79% at concentrations ranging from 0.25 to 4 mg/mL. This finding suggests that MUM212 extract has the capability to scavenge the O_2_^∙-^ produced from the hypoxanthine-xanthine oxidase system as reflected by the decrease in absorbance of the yellow water-soluble WST formazan which was formed upon reduction by O_2_^∙-^.

The metal-chelating activity of MUM212 extract was also assessed by measuring its ability to compete with ferrozine for Fe^2+^ which forms complexes with ferrozine that can be quantitated spectrophotometrically. The assay revealed a decrease in absorbance of Fe^2+^-ferrozine complexes after the addition of MUM212 extract. The results implied that the complex formation was disrupted by MUM212 extract which may be due to the presence of constituents that exhibit metal chelating ability. The MUM212 extract was demonstrated significant metal chelating activity measuring from 9.17 ± 1.16 to 41.98 ± 0.73% at concentrations ranging from 0.25 to 4 mg/mL.

### Effect of MUM212 Extract on Lipid Peroxidation

**FIGURE 3:**
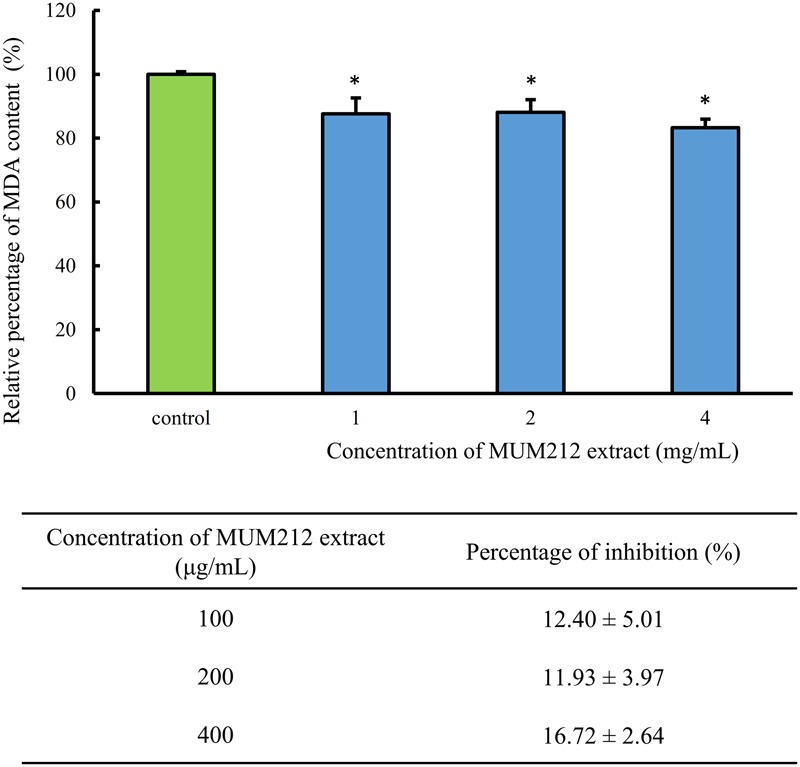
**Effect of MUM212 extract on the MDA level in egg homogenate added with Fe^2+^.** MDA level was quantified by TBARS assay. All data are presented as the mean ± SD (*n* = 3). ^∗^Denotes *p* < 0.05 between control sample (without extract) and MUM212 extract treated sample.

Lipid peroxidation is one of the earliest known and most extensively studied manifestations of oxygen toxicity in biology. Lipid peroxidation involves a chain reaction of oxidative destruction induced by free radicals especially on polyunsaturated fatty acids, resulting in the generation of toxic and mutagenic byproducts. MDA appears to be the most mutagenic product and has been used as a biomarker for lipid peroxidation for many years (Marnett, 1999). Thus, we assessed the effects of MUM212 extract on non-enzymatic peroxidation induced by ferrous sulfate in egg yolk homogenate. TBARS assay was used to quantify the MDA level generated by iron-induced non-enzymatic peroxidation in the lipid-rich homogenate. In **Figure 3**, MUM212 extract at all concentrations tested were shown to inhibit lipid peroxidation as demonstrated by the significant reduction (*p* < 0.05) in the relative percentage of MDA level as compared to the control group (without extract). The result demonstrated that the incubation of MUM212 extract at 4 mg/mL with Fe^2+^ containing sample resulted in 16.72 ± 2.64% inhibition of the extent of lipid peroxidation.

### Cytoprotective Effect against Oxidative Damage Induced by H_2_O_2_

**FIGURE 4:**
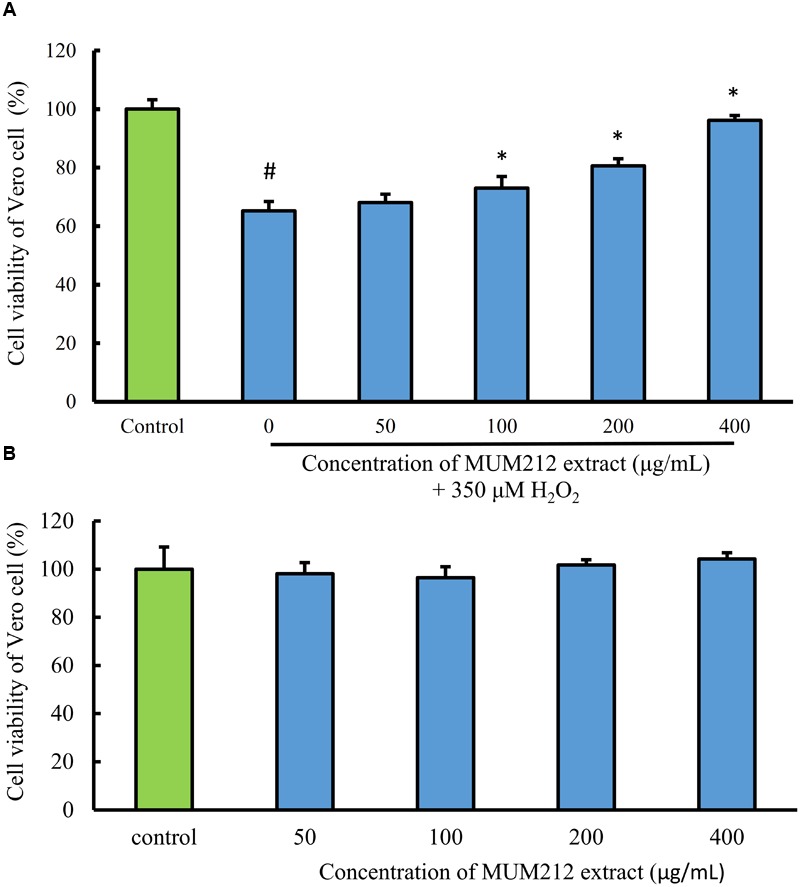
**(A)** Cell protective effect of MUM212 extract on H_2_O_2_-induced cytotoxicity on Vero cells. Cells were pretreated with 50–400 μg/mL of MUM212 extract for 2 h and then exposed to 350 μM H_2_O_2_ for 24 h. The viability of control cells (without MUM212 extract and H_2_O_2_) was defined as 100%. Data shown are mean ± SD (*n* = 5). # Denotes *p* < 0.05 between control cells and H_2_O_2_ only treated cells. ^∗^Denotes *p* < 0.05 between H_2_O_2_ only treated cells and MUM212 extract pretreated cells. **(B)** Cytotoxicity of MUM212 extract on Vero cells. Cells were treated with 50–400 μg/mL of MUM212 extract for 24 h.

To determine whether MUM212 extract can protect cells from oxidative stress, the effect of MUM212 extract on the cytotoxicity of H_2_O_2_ toward Vero cells was examined. The cell viability of Vero cells, measured by MTT assay, was decreased to 65.2% in the control group after exposure to 350 μM H_2_O_2_ for 24 h. Meanwhile, the pretreated Vero cells with MUM212 extract (100–400 μg/mL) were exhibiting higher viability value than the cells without pretreatment of MUM212 extract followed by the exposure to 350 μM H_2_O_2_ (**Figure 4A**). The treatment of Vero cells with MUM212 extract at these concentrations alone did not cause any significant effect on the cell viability (**Figure 4B**). These result suggested that the H_2_O_2_-induced cytotoxic effects on Vero cells were attenuated in the presence of MUM212 extract, demonstrating protective effect against oxidative damages.

### Phenolic and Flavonoid Contents of MUM212 Extract

The TPC of MUM212 extract was estimated using the Folin–Ciocalteu’s reagent method. The Folin–Ciocalteu’s reagent method estimates the TPC of MUM212 extract based on the measurement of the total concentration of phenolic hydroxyl group which reacts with Folin–Ciocalteu’s reagent to form blue complexes in the extract. The result showed that detection of blue complexes as measured by the absorbance at 750 nm was increased with increasing concentrations of MUM212 extract, suggesting the presence of phenolic compounds in the extract. Meanwhile, the flavonoid content determination assay showed negative result from the spectrophotometric measurement, indicating that there were no flavonoids in the MUM212 extract or that they were present in concentrations too low for assay to detect.

A correlation analysis was also performed to assess the relationship between the antioxidant capacity of MUM212 extract and its TPC. The Pearson’s correlation coefficients between the variables are presented in **Table 4**. The Pearson correlation analysis indicated that the highest positive significant correlation was between the TPC and ABTS scavenging activity of MUM212 extract with (*r* = 0.995, *p* < 0.05). The analysis suggested that the antioxidant capacity of MUM212 extract was largely contributed by the phenolic compounds present in the extract as reflected by the strong positive correlation between TPC and all the antioxidant properties tested.

**Table 4:** Pearson’s correlation coefficients between TPC and antioxidant activities of MUM212 extract.

Antioxidant activities	Phenolic content
DPPH radical scavenging activity	*r* = 0.935^∗^
ABTS radical scavenging activity	*r* = 0.995^∗^
SOD-like activity	*r* = 0.847^∗^
Metal-chelating activity	*r* = 0.962^∗^

*^∗^Correlation is significant at the 0.05 level.*

### Chemical Profiling of Streptomyces MUM212 Extract Using GC–MS Analysis

**FIGURE 5:**
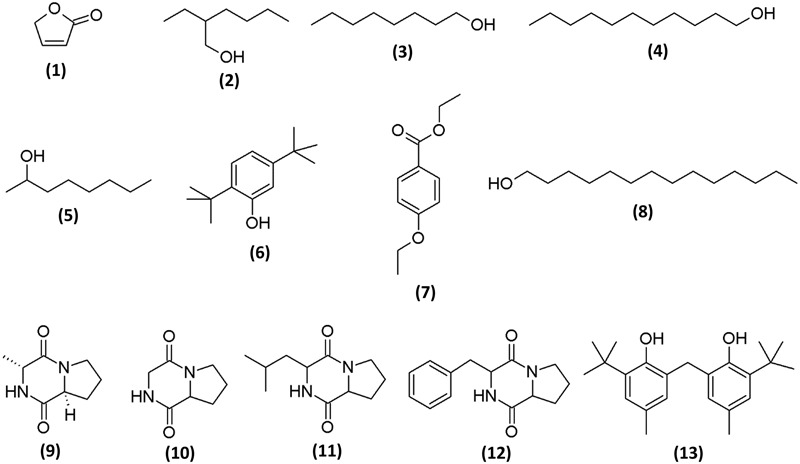
**Chemical structures of constituents detected in MUM212 extract**.

To determine the chemical constituents that may be responsible for its the antioxidant properties, MUM212 extract was subjected to GC/MS analysis. In the present study, GC/MS analysis successfully detected esters, alcohols, phenols and cyclic dipeptides in the complex mixtures of MUM212 extract. These chemical compounds were identified by comparison of their mass spectra to the database available on the W9N11 MS library. The detailed information of the chemical compounds based on their retention time, molecular weight and molecular formula are listed in **Table 5** and their chemical structures are depicted in **Figure 5**.

**Table 5:** Chemical constituents identified in MUM212 extract.

No.	Constituents	Retention time (min)	Molecular formula	Molecular weight (MW)	Similarity (%)
1	2(5H)-furanone	13.913	C_4_H_4_O_2_	84	83
2	2-Ethyl hexan-1-ol	21.472	C_8_H_18_O	130	83
3	1-Octanol	27.296	C_8_H_18_O	130	83
4	1-Undecanol	27.325	C_11_H_24_O	172	90
5	2-Octanol	27.829	C_8_H_18_O	130	83
6	Phenol, 2,5-*bis*(1,1-dimethylethyl)	44.445	C_14_H_22_O	206	95
7	Ethyl 4-ethoxybenzoate	44.897	C_11_H_14_O_3_	194	94
8	1-Tetradecanol	47.529	C_14_H_30_O	214	83
9	(3R,8aS)-3-Methyl-1,2,3,4,6,7,8, 8a-octahydropyrrolo[1,2-a]pyrazine-1,4-dione-; Cyclo(L-Pro-D-Ala)	51.598	C_8_H_12_N_2_O_2_	168	90
10	Pyrrolo[1,2-a]pyrazine-1,4-dione, hexahydro-; Cyclo(Gly-Pro)	53.125	C_7_H_10_N_2_O_2_	154	96
11	Pyrrolo[1,2-a]pyrazine-1,4-dione, hexahydro-3-(2-methylpropyl)-; Cyclo(Leu-Pro)	59.368	C_11_H_18_N_2_O_2_	210	72
12	Pyrrolo[1,2-a]pyrazine-1,4-dione,hexahydro-3-(phenylmethyl)-; Cyclo(Pro-Phe)	72.048	C_14_H_16_N_2_O_2_	244	83
13	Phenol, 2,2′-methylene*bis*[6-(1,1-dimethylethyl)-4-methyl-]	73.507	C_23_H_32_O_2_	340	94

## Discussion

The mangrove forest, located along the intertidal of tropical and subtropical coastal regions, is a woody plant area that exist in conditions of high salinity, extreme tides, strong winds, high temperatures with muddy, anaerobic soils (Kathiresan and Bingham, 2001). It plays an important role in coastline protection and represents one of the world’s most prolific environments rich in supply for forest products and coastal fisheries. It creates unique environments that host a rich assemblages of species, particularly, the muddy or sandy sediments of mangrove forest are favorable habitats for a great diversity of marine, freshwater and terrestrial flora and fauna, and microorganisms (Kathiresan and Bingham, 2001; Jennerjahn and Ittekkot, 2002). Living at the interface between land and sea, the natural stressors (constant fluctuations of salinity, tidal gradient, temperature) (McKee, 1995) are believed to be the driving forces for metabolic pathway adaptations of the microorganisms which live in that environment for survival, leading to production of unique bioactive metabolites (Hong et al., 2009; Lee et al., 2014c). Among these microorganisms, *Streptomyces* sp. have been identified as potential producers of metabolites with interesting biological activities useful to man (Conda-Sheridan et al., 2010; Kondratyuk et al., 2012; Yuan et al., 2013; Ser et al., 2016b). Therefore, the exploration of *Streptomyces* sp. from unexplored environments such as mangrove sediments is one of the most efficient approaches for the discovery of potential novel bioactive metabolites. In the present study, strain MUM212 was isolated from a soil sample collected from an underexplored mangrove forest in Selangor, Malaysia. This study also further explored the bioactive potential of strain MUM212 by evaluating its potential in the production of antioxidative agents. It was suggested that the microbes living in that mangrove region may have acquired the ability to synthesize metabolites high in antioxidant activity or develop specific antioxidant defense mechanisms for survival against oxidative stress (Hong et al., 2009; Lee et al., 2014c).

The strain identified as MUM212 belongs to the genus *Streptomyces.* It was well-characterized through the employment of phylogenetic analysis based on 16S rRNA gene sequences. According to the results of phylogenetic analysis, strain MUM212 shares 99.5% 16SrRNA sequence similarity to that of *S. misionensis* NBRC13063^T^ and followed by *S. phaeoluteichromatogenes* NRRL5799^T^ at 99.4% sequence similarity. Phenotypically, strain MUM212 possesses vivid yellow aerial mycelium and moderate yellow vegetative mycelium. It also forms spiral spore chains which can be observed via scanning electron microscopy. This study also characterized the strain MUM212 in term of its physiological and biochemical properties in order to further improve the understanding of the strain. Strain MUM212 has the potential to produce industrially important enzymes such as amylase and cellulase, which are in great demand in the industrial sectors. Furthermore, strain MUM212 is able to tolerate salinity of 6% NaCl concentration and temperatures up to 40°C, which is unsurprising as these characteristics are essential for the survival of strain MUM212 in the harsh and fluctuating growth conditions found in its natural habitat.

The availability of carbon and nitrogen sources is essential to the production of secondary metabolites in *Streptomyces* sp. whereby the types and quantity of the metabolites are influenced greatly by the composition of substrates given during the growth of *Streptomyces* sp. (Ser et al., 2016a). Thus, the present study utilized the Biolog GEN III MicroPlate system to investigate the carbon and nitrogen utilization of strain MUM212 in order to gain an overview of its metabolite profile. The results revealed the capability of strain MUM212 to utilize a wide range of carbon and nitrogen sources, such as polysaccharides (pectin and dextrin), monosaccharides (α-D-glucose, D-fructose, D-galactose), glycosides (*N*-acetyl-D-glucosamine), amino acids (L-alanine, L-histidine, and L-serine) and sugar alcohols (D-mannitol and D-arabitol). This data would potentially have key applications in future work regarding medium optimization for higher yield of the desirable bioactive compounds.

Given that the process of oxidation is very complex and may occur via multiple mechanisms, a single antioxidant assay is insufficient to assess the total antioxidant capacity of an extract from natural products (Apak et al., 2016). This is partly due to the presence of various types of antioxidants in these substances including various polyphenols, reducing agents and metal chelators all of which elicit antioxidative effects via different mechanism of actions (Brewer, 2011). Hence, multiple antioxidant assays are needed to assess the overall antioxidant capacity of an extract, with the assays selected based on the consideration of functions to be evaluated. A total of four antioxidant assays were utilized in this study to evaluate the antioxidant properties of MUM212 extract. These antioxidant assays demonstrated that the MUM212 extract exhibits various antioxidant activities including DPPH radical scavenging activity, ABTS radical scavenging activity, SOD-like activity and metal chelating activity.

Both DPPH and ABTS assays are classified as single electron transfer reactions (Apak et al., 2016) and are used to assess the antioxidant reductive capacity of the test extract to neutralize both of these radical indicators either by direct reduction through electron transfer or by radical quenching by hydrogen transfer. Although both the DPPH and ABTS systems are not biologically relevant, both of these assays are robust and simple to conduct. This study thus employed these assays as preliminary screening to estimate the antioxidant capacity of the extract based on its radical scavenging activities on different free radicals. Besides that, the MUM212 extract was shown to exhibit SOD-like activity, suggesting that it may help in preventing the excessive generation of O_2_^∙-^ which is a key event in many pathological events such as carcinogenesis (López-Lázaro, 2007). Furthermore, the excessive generation of O_2_^∙-^ can result in the formation of highly reactive peroxynitrite molecules (ONOO^-^) through the reaction between O_2_^∙-^ and nitric oxide or alternatively lead to higher production of H_2_O_2_ which can result in the non-enzymatic production of the notorious hydroxide radical (OH^∙^) in the presence of Fe^2+^ through Fenton reaction (Dunford, 2002; Imlay, 2008). Thus, the control of O_2_^∙-^ generation is of great importance to prevent redox imbalance due to the increased production rate of O_2_^∙-^ that overwhelms the capacity of the endogenous SOD enzyme defense system to prevent the free radicals from harming the cells.

The Fenton reaction involves the chemical decomposition of hydrogen peroxide in the presence of iron as the catalyst (Lin and Gurol, 1998), resulting in the production of ROS such as the highly reactive hydroxyl radicals which can cause damage to molecules including lipids, proteins and DNA (Prousek, 2007). Furthermore, transition metals also accelerate lipid peroxidation by decomposing lipid hydroperoxides into hyperoxyl and alkoxyl radicals which can lead to the production of the carcinogenic and mutagenic electrophile MDA that form adducts with DNA bases (Hazra et al., 2008). Over time, this process results in cell death, mutagenesis, and subsequently carcinogenesis. Therefore, the metal chelating capacity as demonstrated by MUM212 extract is significant since it may aid in inhibiting the process of lipid peroxidation by stabilizing the catalytic transition metals. Indeed, MUM212 extract was shown to inhibit lipid peroxidation induced by Fe^2+^ as demonstrated by the reduction in MDA levels. MUM212 extract may have prevented the formation of hydroperoxides and the subsequent production of MDA in the lipid rich egg homogenate which is prone to peroxidation in the presence of transition metals. These findings indicate that the MUM212 extract, which exhibits good metal chelating ability, not only inhibits metal-induced oxidative stress, but can also be a good source for preventive approach against ROS-mediated diseases.

To further support the antioxidant potential of MUM212 extract, this study employed an *in vitro* bioassay to evaluate the antioxidative effect of the extract in an oxidative stress-induced cellular model. Interestingly, MUM212 extract was demonstrated to confer a protective effect against ROS induced oxidative damage in cells. H_2_O_2_ was used as an inducer of oxidative stress in the *in vitro* cellular model of normal cell line. The H_2_O_2_ molecule has high cell membrane permeability which passes through the cell membrane readily and results in production of highly reactive hydroxyl radicals in the cells which can damage the intracellular macromolecules including the protein, DNA and lipids, ultimately leading to cell death (Hampton and Orrenius, 1997). Furthermore, H_2_O_2_ is also a major component of ROS produced intracellularly and a cause of oxidative damage (Finkel and Holbrook, 2000). Oxidative stress has been associated with many pathological manifestations of human diseases such as cancer, diabetes, cardiovascular, and neurodegenerative diseases. In this study, MUM212 extract was shown to attenuate the cytotoxicity induced by H_2_O_2_ on the Vero cells, in which pre-treatment of MUM212 extract restored the cell survival of Vero cells exposed to H_2_O_2_. This result suggests that MUM212 extract is capable of protecting Vero cells from oxidative stress-induced cellular injuries. Based on the overall results, MUM212 extract appears to be a good resource for the development of effective dual functioning antioxidants, exhibiting both free-radical scavenging and metal-chelating properties. MUM212 extract may have the potential to be developed into antioxidative drugs which are effective in protecting against oxidant induced cellular damage.

The strong correlation between the four assays measuring antioxidant capacity and the TPC suggests that phenolic compounds make a large contribution to the antioxidant properties of MUM212 extract. Phenolic compounds are characterized by the presence of one or several phenol groups in which some of the phenolic compounds exhibit potent antioxidant activity including scavenging free radicals and also chelating metal ions (Lü et al., 2010). Phenolic compounds have been widely recognized as potent preventive agents, acting as antioxidants and modulators of intracellular signaling processes involved in initiation/promotion of cancer (Soobrattee et al., 2006). However, further characterization of the extract is required in order to verify that the antioxidant capability of MUM212 extract is mainly due to the presence of phenolic compounds. We pursued this via GC/MS, a powerful analytical tool which combines both gas chromatographic separation and mass spectrometric detection for molecular identification in drug discovery; a technology which has already contributed significantly to the investigation of bioactive compounds derived from *Streptomyces* bacteria (Schöller et al., 2002; Wang et al., 2013; Citron et al., 2015; Selvakumar et al., 2015; Ser et al., 2015a).

Based on the results of GC/MS analysis, MUM212 extract was shown to contain chemical compounds including alcohols, esters, phenolics, and cyclic dipeptides. Some of the detected compounds have been reported previously in microbial fermentation broths, including those from actinomycetes and *Streptomyces* sp. For instance, 2(5H)-furanone **(1)** (Braun et al., 1995), 2-ethyl hexan-1-ol **(2)** (Sunesson et al., 1997), 1-octanol **(3)** (Gu et al., 2007; Chaves-López et al., 2015), 1-undecanol **(4)** (Liu et al., 2015), 2-octanol **(5)** (Spreafico et al., 1997), phenol, 2,5-*bis*(1,1-dimethylethyl)- **(6)** (Zhang et al., 2013; Nandhini, 2015), ethyl 4-ethoxybenzoate **(7)** (Sharma et al., 2010), 1-tetradecanol **(8)** (Nhi-Cong et al., 2010), cyclo(L-Pro-D-Ala) **(9)**, cyclo(Gly-Pro) **(10)** (Ser et al., 2015b), cyclo(Leu-Pro) **(11)**, cyclo(Pro-Phe) **(12)** (Jog et al., 2014; Sharma et al., 2016) and phenol, 2,2′-methylene*bis*[6-(1,1-dimethylethyl)-4-methyl-] **(13)** (Tan et al., 2015b).

The phenol,2,5-*bis*(1,1-dimethylethyl)- **(6)** and phenol, 2,2′-methylene*bis*[6-(1,1-dimethylethyl)-4-methyl-] **(13)** were the phenolic compounds detected in the MUM212 extract. The detection of these phenolic compounds in the extract by GC–MS analysis was in line with the results of TPC estimation by the Folin–Ciocalteau’s reagent method which originally suggested the presence of phenolic compounds in the extract. Notably, phenolic compounds are widely regarded as potent antioxidant agents or free radical scavengers, as they possess hydrogen-donating ability to reduce free radicals (Sulaiman et al., 2011; Yogeswari et al., 2012). Previous studies also have evidenced that the presence of phenol,2,5-*bis*(1,1-dimethylethyl)- **(6)** and phenol, 2,2′-methylene*bis*[6-(1,1-dimethylethyl)-4-methyl-] **(13)** in the extracts of *Streptomyces* sp. exhibiting antioxidant properties (Ser et al., 2015a,b. Thus, it is possible that these phenolic compounds play a major role in the antioxidant capacity demonstrated by MUM212 extract, which displays several free-radical scavenging and metal-chelating activities.

Several cyclic dipeptides or 2,5-diketopiperazines (DKP) have been detected from the extract in the present study, including cyclo(L-Pro-D-Ala) or (3R,8aS)-3-methyl-1,2,3,4,6,7,8,8a-octahydropyrrolo[1,2-a]pyrazine-1,4-dione **(9)**, cyclo(Gly-Pro) or pyrrolo[1,2-a]pyrazine-1,4-dione, hexahydro- **(10)**, cyclo(Leu-Pro) or pyrrolo[1,2-a]pyrazine-1,4-dione, hexahydro-3-(2-methylpropyl)- **(11)** and cyclo(Phe-Pro) or pyrrolo[1,2-a]pyrazine-1,4-dione,hexahydro-3-(phenylmethyl)- **(12)**. Cyclic dipeptides or DKP are a group of the simplest peptide derivatives commonly occurring in nature (Prasad, 1995). Recent studies also reported the presence of cyclic dipeptides in the fermentation culture of microbes (Würth et al., 2014; Vázquez-Rivera et al., 2015; Ser et al., 2016b). These cyclic dipeptides compounds identified had been suggested to possess potent antioxidant activity (Ser et al., 2015b; Tan et al., 2015b). There was also evidence of a cyclic dipeptide that exhibits protective effect against oxidative stress in H_2_O_2_-injured neuronal cells by reducing ROS generation and increasing intracellular glutathione levels (Minelli et al., 2009). As a whole, the chemical compounds detected by GC–MS analysis are well-recognized for their antioxidant properties, suggesting that these constituents could be responsible for antioxidant capacity of *Streptomyces* MUM212 extract. Therefore, this study provides further evidence on the potential of mangrove-derived *Streptomyces* sp. as a promising source for antioxidative agents, including strain MUM212.

## Conclusion

**FIGURE 6:**
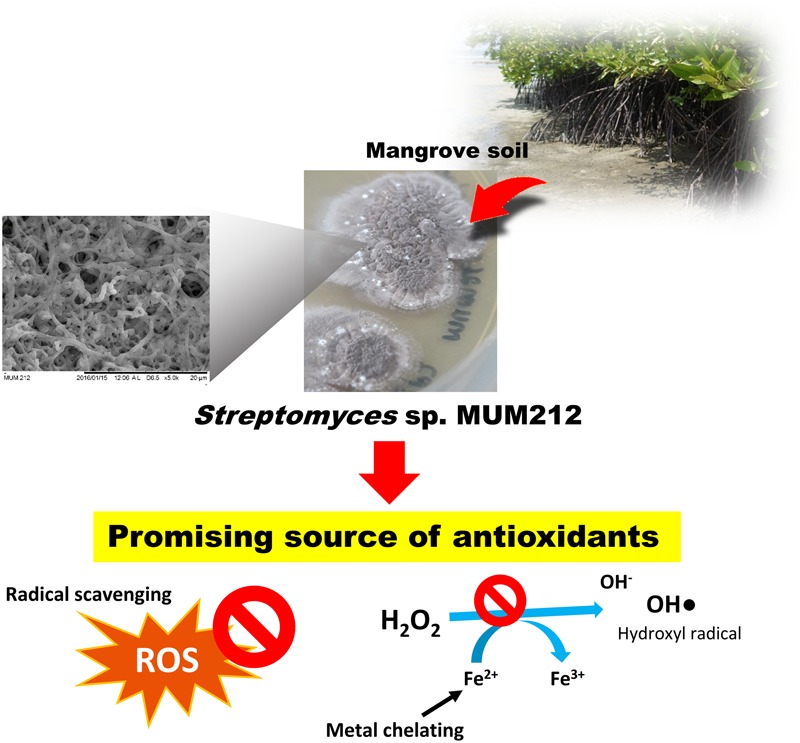
***Streptomyces* sp. MUM212 as a potential source of antioxidants with radical scavenging and metal chelating activities**.

In summary, this study describes the isolation of strain MUM212— a strain with promising antioxidant properties—from mangrove soil. The MUM212 extract is able to scavenge several free radicals including superoxide anion, DPPH and ABTS radicals and also chelate metal ions. The MUM212 extract has the capability to inhibit lipid peroxidation and rescue cells from oxidative-stress induced cellular injuries. The phenolic compounds and cyclic dipeptides present in the extract could be the major constituents responsible for the antioxidant properties of MUM212 extract. Therefore, the findings of the study suggest that the mangrove-derived *Streptomyces* sp. MUM212 could be a potential source of antioxidants with radical scavenging and metal chelating activities (**Figure 6**). Overall, mangrove derived *Streptomyces*, in particular strain MUM212 holds promise for large scale production of antioxidative agents that is of great importance for the development of therapeutic drugs in the intervention of oxidative stress-mediated diseases. MUM212 extract merits further investigations on the intracellular molecular mechanisms of action which, while still elusive, are in fact already in progress.

## Author Contributions

The experiments, data analysis and manuscript writing were conducted by LT, L-HL, and B-HG. B-HG, L-HL, and K-GC contributed by providing vital technical support for the project and PP, SS, AD, TK proofread on the writing. L-HL, K-GC, SB, and B-HG contributed to the funding of the project. L-HL and B-HG founded the research project.

## Conflict of Interest Statement

The authors declare that the research was conducted in the absence of any commercial or financial relationships that could be construed as a potential conflict of interest.

## Footnotes

1 http://www.bacterio.cict.fr/

2 http://www.ezbiocloud.net
