# Supplementary material for: Streptomyces sp. MUM212 as a Source of Antioxidants with Radical Scavenging and Metal Chelating Properties
Source: Front Pharmacol. 2017 May 17;8:276. doi: 10.3389/fphar.2017.00276 (PMC5434116; doi:10.3389/fphar.2017.00276)
Supplement: Supplementary file 1 [file Image_1.PDF]

Figure S1. Colony characteristics of strain MUM212 on different agar plate

Top view

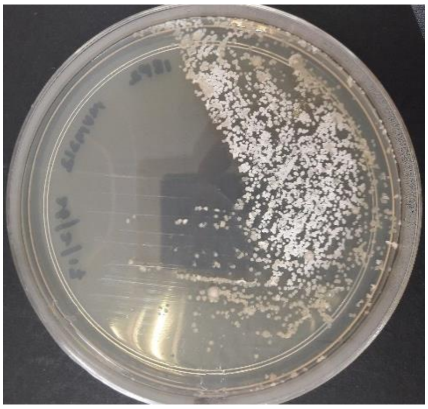

Bottom view

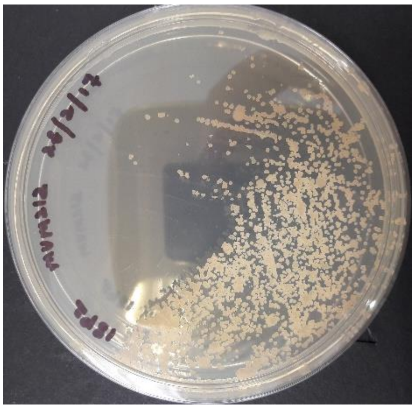

ISP2 agar plate

Top view

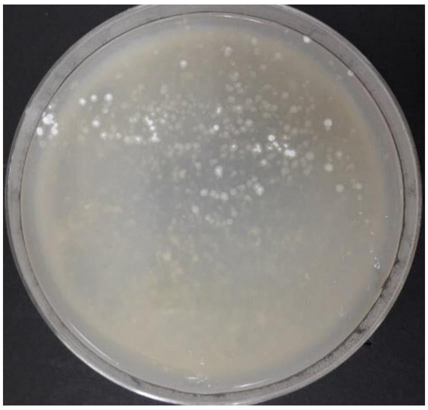

Bottom view

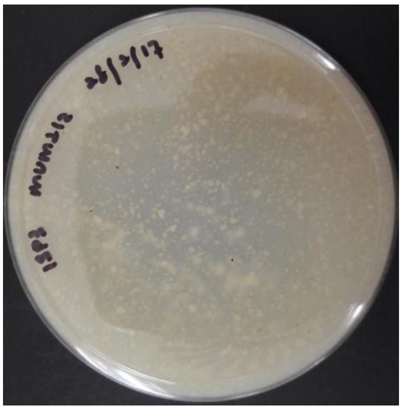

ISP3 agar plate

Top view

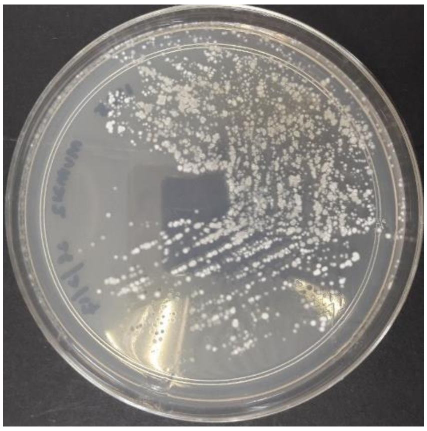

Bottom view

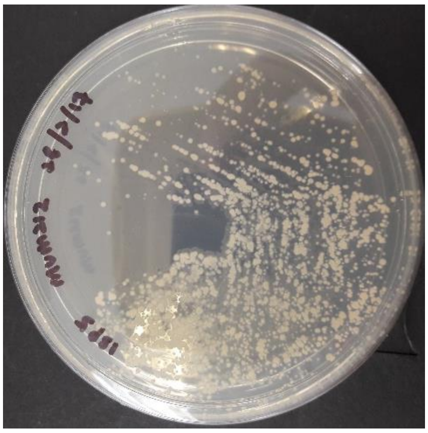

ISP5 agar plate

Top view Bottom view

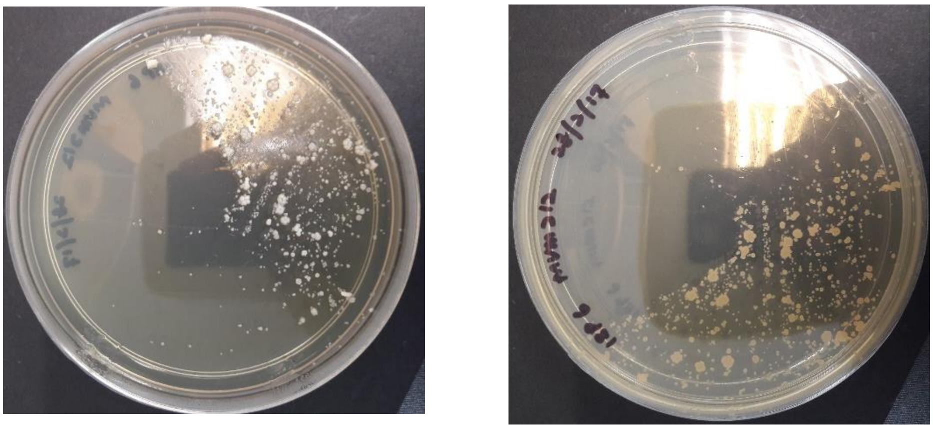

ISP6 agar plate

Top view Bottom view

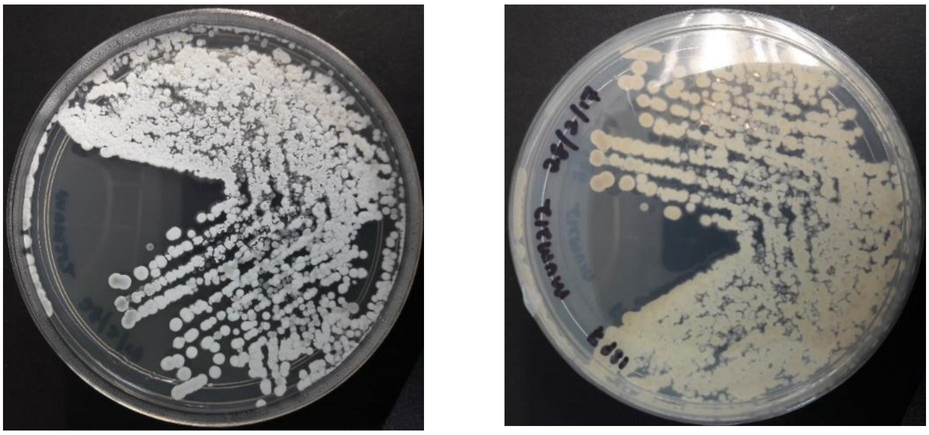

ISP7 agar plate

Top view Bottom view

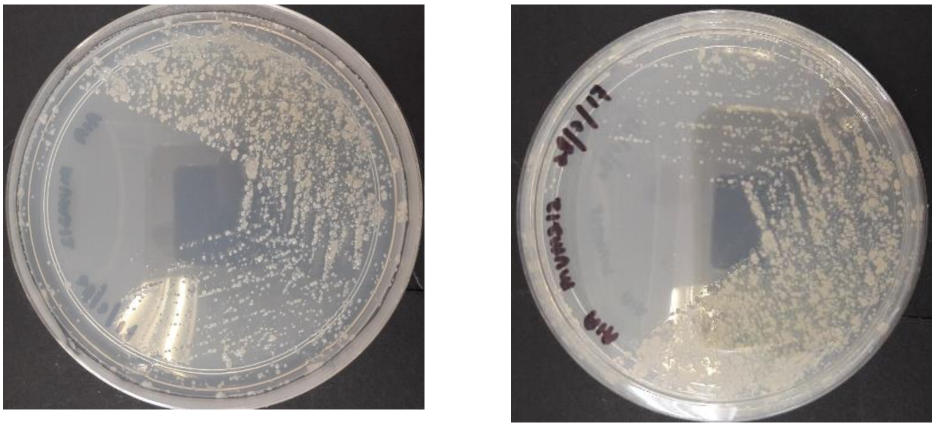

AIA agar plate

Top view

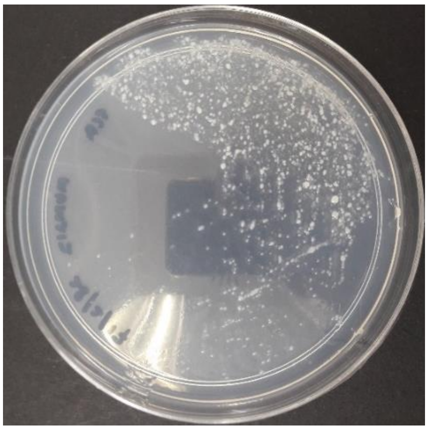

Bottom view

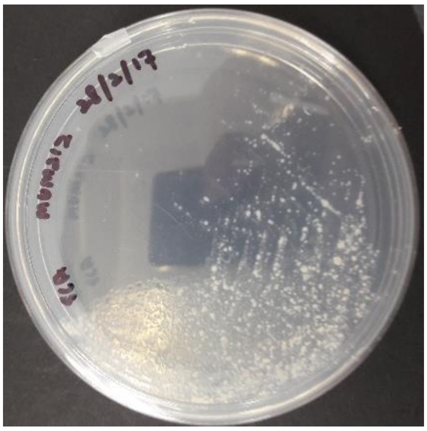

SCA agar plate

Top view

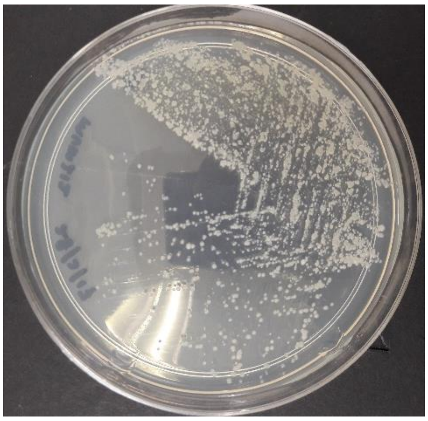

Bottom view

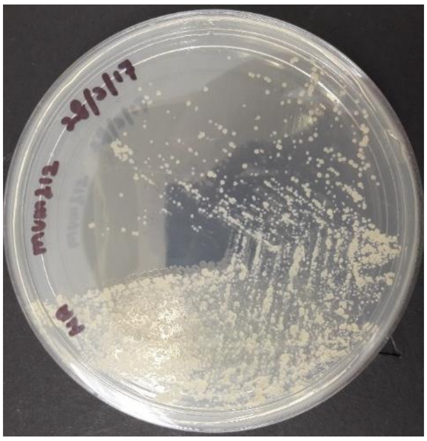

NA agar plate

Figure S2. The enzymatic activities of strain MUM212

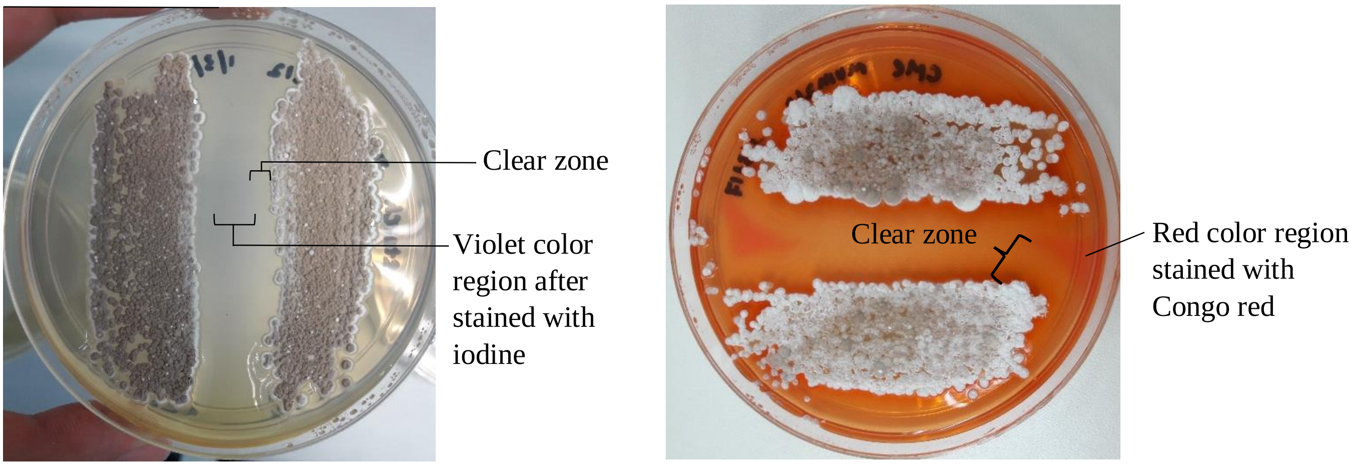

(a) (b)

Strain MUM212 was grown on ISP2 agar plate supplemented with 0.2% (w/v) starch (a) and 0.5% (w/v) CMC (b). In (a), the clear zone indicates strain MUM212 able to digest starch. In (b), the clear zone indicates strain MUM212 able to digest CMC.
